# Relationship between echocardiographic characteristics and cardiac biomarkers during long-distance trail running

**DOI:** 10.3389/fcvm.2022.954032

**Published:** 2022-08-16

**Authors:** Romain Jouffroy, Hélène Hergault, Juliana Antero, Antoine Vieillard Baron, Nicolas Mansencal

**Affiliations:** ^1^Intensive Care Unit, Ambroise Paré Hospital, Assistance Publique—Hôpitaux de Paris (AP-HP), Université de Versailles-Saint Quentin (UVSQ), Boulogne-Billancourt, France; ^2^IRMES - Institute for Research in Medicine and Epidemiology of Sport, INSEP, Paris, France; ^3^INSERM U-1018, CESP, Clinical Epidemiology, Université de Versailles-Saint Quentin (UVSQ), Villejuif, France; ^4^Department of Cardiology, Ambroise Paré Hospital, AP-HP, Centre de Référence des Cardiomyopathies et des Troubles du Rythme Cardiaque Héréditaires ou Rares, Université de Versailles-Saint Quentin (UVSQ), Boulogne-Billancourt, France

**Keywords:** echocardiographic, biomarker, running, long distance, relation

## Abstract

**Background:**

Even if the beneficial cardiovascular effects of moderate exercise are recognized, effects of prolonged and intense exercise are still debated. This study aims to detect cardiovascular changes associated with long endurance running by assessing the relationship between echocardiographic parameters and cardiac biomarkers during long-distance trail running.

**Methods:**

We performed a prospective observational study that included 20 participants who were all amateur runners (median age of 41 years old, still alive after a 7-year clinical follow-up) from 80-km trail running. All the participants underwent an echocardiographic examination and venous blood sampling before the race, at the intermediate refreshment checkpoints of the race (21st and 53rd km), and within 10 min after arrival.

**Results:**

Mitral E/A velocity ratio and mitral TDI e’ wave were significantly decreased at the 21st km to arrival (*p* < 0.05). Mitral S wave and global longitudinal strain (GLS) were significantly decreased from the 53rd km to arrival (*p* < 0.05 for 53rd and 80th km). As compared to baseline, T-troponin and NT-proBNP were significantly increased at the 21st km in all the participants, but T-troponin values were systematically increased above the significative threshold. Diastolic echocardiographic abnormalities were mainly observed among participants with highest NT-proBNP (> 77 ng.l^–1^) values at the 21st km. As compared to baseline, mitral e’ wave was significantly decreased (–35%) in participants with highest values of NT-proBNP. Similarly, GLS was also depressed among participants with highest troponin values at the 53rd km (*p* = 0.01 for 53rd km and *p* = 0.04 for arrival).

**Conclusion:**

During the long-distance trail running, the early LV decrease in diastolic echocardiographic parameters is associated with increase in NT pro-BNP blood levels, and the decrease in LV systolic echocardiographic parameters later is associated with increase in T-troponin blood levels.

## Introduction

In the last 10 years, long-distance running races (i.e., races of at least 6 h) (1) became more popular all over the world (2). This increase of practice has also been observed among individuals older than 45 years old, currently representing around 50% of the participants in marathons and long-distance running races (3). Beneficial cardiovascular effects of moderate exercise are recognized (4), while cardiovascular effects of prolonged and intense exercise remains debated (5–7). Physical activity such as long-term endurance sport practice induces a cardiac remodeling named “athlete’s heart”(8–10), mainly concerning the left ventricle (LV), but also right ventricle (RV) by inducing right ventricular remodeling (11), even if clinical significance is unclear (12, 13). We have previously found that echocardiographic LV systolic and diastolic abnormalities may occur in amateur ultra-distance trail runners (14), and may also impact the RV, because intense exercise-induced right ventricular remodeling is a potential adaptation of cardiac function and structure.

In myocardial injury and septic shock, troponin levels are closely correlated with myocardial injury (15–17). Troponin elevation may result from several mechanisms: parietal mechanical stress in response to pressure or volume overload (18), tachycardia (19), massive release of catecholamines (20), direct action of PAMPs (pathogen-associated molecular patterns) or lipopolysaccharides in sepsis (21), or viral, bacterial, or immune myocarditis (22). Previous studies have found a significant increase in cardiac biomarkers [N-terminal pro-brain natriuretic peptide (NT-proBNP) and troponin] during long-distance exercises (23, 24), with unclear significance (25, 26). Recently, it has been observed that a troponin exercise-induced increase above the 99th percentile could be an early marker of mortality and cardiovascular events (27) notably in participants with hypertension (28). Nevertheless, these results remain to be discussed (7, 26, 29, 30). To date, no study has reported a relationship between echocardiographic characteristics and cardiac biomarkers in long distance runners. We aim to detect cardiovascular changes associated with long endurance running by assessing the relationship between echocardiographic parameters and cardiac biomarkers during long-distance trail running.

## Materials and methods

### Study population and study protocol

We performed a prospective observational study on 20 participants who were all amateur runners in the 80-km Ecotrail of Paris Ile de France© (total climb of 1,500 m with 4 refreshment points). All the participants were electronically recruited by announcement on the race’s website.^1^ The protocol was approved by the race’s organization committee, all the participants, the French committee on public safety Paris Ile-de-France IV (Reference: 2014/07), and the National Heart Agency (Number EudraCT: 2014-A00205-42).

All participants who fulfilled the following criteria were included before the race event: age > 18 years, male gender, and previous completion of an ultra-endurance race (distance > 50 km) during the last 12 months. Participants with a medical history (hypertension, cardiomyopathy, heart failure, or valvular heart disease) or with abnormalities detected on the first echocardiographic examination were not included in the study.

### Echocardiographic examination by transthoracic echocardiographic examination

All the participants underwent a baseline echocardiographic examination (< 24 h of the start of the event) to determine morphological and dynamical echocardiographic parameters and to confirm the inclusion of subjects. Echocardiographic examinations were performed, and venous blood sampling was systematically performed for sample collection before the race, at the intermediate refreshment checkpoints of the race (21st and 53rd km), and within 10 min after arrival. During the race, a 5-min stop at each checkpoint was required for echocardiographic recording and blood sampling.

All the echocardiographic examinations were performed and recorded by a single experienced physician (NM) using VIVID I (GE Medical Systems, Horten, Norway) (14).

### Blood sampling

Blood samples were drawn (1 ml/sample), collected, and immediately stored on ice by a nurse in order to be analyzed later. Electrolytes (Na, K, Cl, and HCO3-), creatinine, and urea plasma concentrations, high-sensitivity T-troponin, NT-proBNP, creatine kinase (CK), myoglobin, protein, and C reactive protein (CRP) blood levels were measured with the immunochemiluminescence method (Roche Diagnostics©) within 2 weeks after the end of the race in a central laboratory of a hospital in Paris (France). In accordance with the laboratory’s biological standards, the normal value for troponin was < 14 ng.l^–1^ and the normal value for NT-proBNP was < 300 ng.l^–1^.

All the echocardiographic examinations were digitally recorded and independently interpreted by a physician blinded to the clinical and biological status and race results. After the race, all the measurements were performed according to recommendations (30) and averaged over 3 cardiac cycles. Several 2-dimensional (2D) views were consistently recorded: parasternal long-axis view, parasternal short-axis view, and apical 2-, 3-, and 4-chamber views. The following 2D measurements were systematically assessed (13): (1) end diastolic measurements of the interventricular septum and posterior wall of the LV, (2) end systolic LV and left atrial diameters, (3) aorta diameter and aortic outflow tract in the parasternal long-axis view, (4) LV ejection fraction using Simpson’s method, and (5) left atrial diameter. The following Doppler parameters were consistently calculated: (1) E and A waves of mitral inflow and mitral E wave deceleration time. (2) aortic ejection flow (peak of aortic ejection flow and velocity-time integral of aortic flow), allowing for measurement of cardiac output, (3) Doppler tissue imaging of the lateral and septal mitral annuli in the apical 4-chamber view, allowing for average measurement of mitral e’, a’, and S waves and of the E/e’ ratio, and (4) Doppler tissue imaging of the lateral tricuspid annulus in the apical 4-chamber view, allowing for measurement of tricuspid e’, a’, and S waves. Regional and global LV functions were also studied using the longitudinal strain assessed by 2D speckle tracking. For the analysis of myocardial deformation, loops were recorded in standard B mode using 70–80-Hz cadence images. The software tracks the positional changes of natural myocardial acoustic markers. We systematically calculated global longitudinal strain (GLS) from the apical 2-, 3-, and 4-chamber views.

### Statistical analysis

Continuous variables were expressed by median and interquartile range (1st quartile–3rd quartile). Categorical data were expressed as absolute value and percentage.

First, bivariate analyses were performed to assess the relationship between each covariate value on km 21, 53, and 80 (within 10 min after the end of the race) compared with baseline value (km 0). Comparisons were performed by paired data analysis based on the Wilcoxon rank test to take into account the repeated measure design of the study. Second, a Wilcoxon-test was performed between significant echocardiography systolic and diastolic parameters and troponin and NT-proBNP blood median values. The median value was chosen to assess the relation between echocardiographic measurements and participants above the median value for T-troponin and NT-proBNP at each evaluation point respectively in order to identify a biological threshold for cardiac burn-out.

All the tests were 2-sided with a statistically significant *p*-value < 0.05. All the analyses were performed using R 3.4.2 (the R Foundation for Statistical Computing, Vienna, Austria).^2^

## Results

### Population characteristics

Twenty male participants at the 80-km Ecotrail of Paris Ile de France© 2014 were included. On 14 March 2014, the day of the race, the weather was clear (no rain), and the temperature was 14 degrees Celsius. No participant had a cardiovascular risk factor. The characteristics of the sample population are presented in Table 1.

**TABLE 1:** Characteristics of 20 amateur ultra-distance and trail runners.

Variable	Median	IQR	Min-max values
Age (years)	41	40–46	26–58
Weight (kg)	74	65–79	60–88
Height (cm)	175	172–180	167–190
Body surface area (m^2^)	1.93	1.78–1.98	1.67–2.16
Body mass index (kg.m^–2^)	23.8	22.0–24.8	21.2–24.9
Training (hours.week^–1^)	5	4–6	1–14
Training (km.week^–1^)	50	40–60	10–75
Duration of the race (hours.min)	11.1	9.1–11.8	9.09–12.41
Average speed of the race (km.h^–1^)	7.4	6.6–8.3	6.3–8.8

Results are expressed by median and interquartile (IQR) range (1st–3rd quartile), and minimal (min) and maximal (max) values.

All the participants reached the finish line. They were middle aged (median age of 41 years old), quite slim (median BMI of 23.8 kg.m^–2^), and well-trained with regular practice of running (median of 5 h and 50 km running per weak; Table 1). On 1 January 2022, all the participants were still alive with regular long-distance practice: annual mean of 5 ± 3 long-distance running races.

### Echocardiographic parameters

The evolution of blood parameters from baseline to arrival are presented in Table 2. We observed significant modifications in diastolic function at the 21st km and of systolic function at the 53rd km. Mitral E/A velocity ratio and mitral TDI e’ wave were significantly decreased from 21 to 80 km (*p* < 0.05). Mitral S wave and GLS absolute values were significantly decreased from the 53rd km to arrival (*p* < 0.05 for the 53rd and 80th km), contrary to LVEF (*p* < 0.05 only on the 80th km). All the systolic function parameters were significantly decreased at arrival.

**TABLE 2:** Echocardiographic characteristics of the 20 amateur ultra-distance and trail runners.

Variable	Baseline (*n* = 20)	Km 21 (*n* = 20)	Km 53 (*n* = 20)	Km 80 (*n* = 20)
**End-diastolic measurements**				
Interventricular septum (mm)	9.9 (9.0 ± 10.3)	9.6 (8.9–10.3)*	9.4 (8.8–9.9)	9.5 (8.8–10.0)
Posterior wall (mm)	9.3 (8.0–10.1)	9.2 (8.7–10.2)	9.1 (8.9–10.1)	9.9 (9.1–10.1)
Left ventricular diameter (mm)	48.8 (43.4–51.0)	45.4 (42.3–47.5)	44.2 (41.0–46.1)	44.4 (41.0–48.4)*
Left ventricular ejection fraction (%)	65 (62–66)	66 (65–67)	63 (60–65)	59 (55–62)*
**Doppler measurements**				
Mitral S wave (m.s^–1^)	16.0 (15.0–18.0)	17.0 (13.8–19.3)	15.5 (13.0–17.3)*	14.0 (13.0–15.5)*
Mitral E wave (m.s^–1^)	0.80 (0.75–0.93)	0.77 (0.66–0.84)	0.70 (0.60–0.73)	0.60 (0.53–0.68)*
Mitral A wave (m.s^–1^)	0.61 (0.50–0.66)	0.76 (0.70–0.82)	0.72 (0.61–0.86)	0.62 (0.73–0.73)
Mitral E/A ratio	1.02 (0.99–1.06)	0.98 (0.89–1.07)*	0.94 (0.82–1.10)*	0.88 (0.79–1.40)*
Mitral TDI e’ wave (m.s^–1^)	0.12 (0.11–0.14)	0.10 (0.08–0.13)*	0.09 (0.07–0.14)*	0.09 (0.08–0.12)*
Mitral E/e’ ratio	6.3 (5.5–7.6)	6.8 (6.1–7.4)	6.3 (5.6–7.5)	5.9 (5.4–6.8)
Tricuspid TDI e’ wave (m.s^–1^)	0.16 (0.14–0.17)	0.12 (0.11–0.16)	0.13 (0.11–0.16)	0.13 (0.11–0.14)
Tricuspid E/e’ ratio	5.0 (4.4–5.9)	5.5 (5.0–6.3)	4.7 (4.4–5.9)	5.0 (4.6–5.3)
**Two-dimensional strain measurements**				
Global peak systolic strain (%)	–21.5 (–22.3 to –20.0)	–21.2 (–22.7 to –21.6)	–19.9 (–23.4 to –18.9)*	–19.8 (–20.3 to –18.7)*
A3C peak systolic strain (%)	–21.6 (–23.8 to –20.8)	–22.2 (–25.0 to –21.1)	–20.6 (–24.3 to –19.3)*	–20.6 (–21.6 to –18.9)*
A4C peak systolic strain (%)	–21.0 (–22.8 to –19.3)	–21.9 (–23.4 to –20.7)	–20.1 (–21.1 to –17.9)*	–19.0 (–20.2 to –18.3)*
A2C peak systolic strain (%)	–21.9 (–23.2 to –20.8)	–20.8 (–23.9 to –21.5)	–20.5 (–28.4 to –19.6)	–20.3 (–21.5 to –17.9)*

A2C, apical 2-chamber view; A3C, apical 3-chamber view; and A4C, apical 4-chamber view. *For p-value < 0.05 with baseline.

Results are expressed by median and IQR range (1st–3rd quartile).

### Biological parameters

The evolution of blood parameters from baseline to arrival are presented in Table 3. In comparison with the baseline value, troponin and NT-proBNP were significantly increased at the 21st km in all the participants. While the T-troponin values remained relatively stable, we observed a significant progressive and constant increase in NT-proBNP values during the race. T-troponin values increased above the significative threshold (N < 14 ng.l^–1^) in all the participants at the 21st km contrary to the NT pro-BNP values, which remained below the threshold (N < 300 ng.l^–1^) in all the participants all along the race.

**TABLE 3:** Evolution of biomarkers in the 20 amateur ultra-distance and trail runners.

Variable	Baseline	Km 21 (*n* = 20)	Km 53 (*n* = 20)	Km 80 (*n* = 20)
Sodium (mmol.l^–1^)	143 (141–145)	143 (141–144)	146 (142–150)	139 (137–145)
Potassium (mmol.l^–1^)	4.6 (4.4–5.1)	5.8 (5.1–7.1)	4.8 (4.5–4.9)	4.6 (4.3–5.0)
Urea (mmol.l^–1^)	5.4 (4.5–6.4)	5.8 (5.1–7.1)*	8.2 (7.2–9.2)*	9.4 (7.8–10.6)*
Creatinine (mmol.l^–1^)	88 (81–97)	106 (94–131)*	131 (110–153)*	116 (98–132)*
Proteins (g.l^–1^)	76 (70–82)	77 (76–80)	76 (73–79)	73 (70–74)
C reactive protein (mg.l^–1^)	0 (0–0)	0 (0–0)	1.5 (0–2.1)*	6.2 (3.9–8.3)*
High-sensitivity T-troponin (ng.l^–1^)	8.3 (2.1–12.2)	15.9 (10.8–20.1)*	23.2 (17.4–38.5)*	19.0 (13.9-33.7)*
Brain natriuretic peptide (pg.ml^–1^)	0 (0–0)	0 (0–3.1)	2.7 (0.6–5.0)*	5.8 (2.7–9.7)*
N-terminal pro-brain natriuretic peptide (ng.l^–1^)	19 (16–20)	62 (47–75)*	91 (80–105)*	112 (90–136)*
Myoglobin (microg.l^–1^)	25 (16–31)	170 (138–228)*	1,115 (580–1,942)*	1,428 (989–2,717)*
Creatine kinase (ng.ml^–1^)	14 (9–16)	12 (11–17)	25 (22–30)*	64 (25–72)*

*For p-value < 0.05 with baseline. Results are expressed by median with interquartile range (1st–3rd quartile).

Muscle biomarkers values (myoglobin, creatinine, and CK) were also above the lower normal value at the 21st km and progressively increased until the end of the race. A systemic inflammation appeared at the 53rd km suggested by the significant and progressive increase in CRP, but no significant association with echocardiographic abnormalities was observed. Urea and creatinine were also progressively increased from the 21st km to the end of the race.

### Relationship between echocardiographic parameters and cardiac biomarkers

**FIGURE 1:**
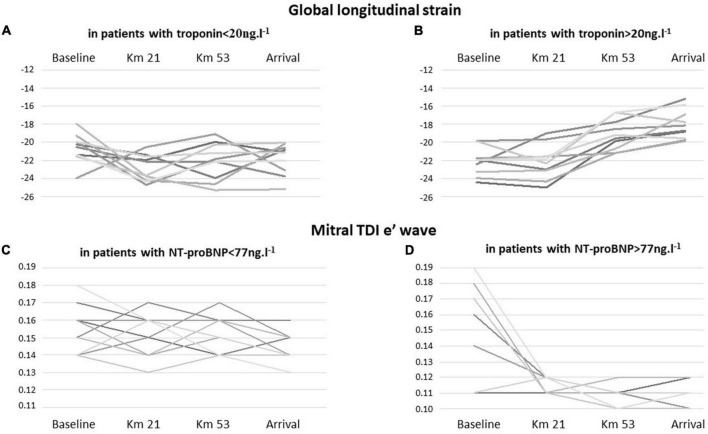
Individual changes in global longitudinal strain in runners according to the results of troponin during the race [**(A)** troponin levels < 20 ng.l^−1^ versus **(B)** troponin levels > 20 ng.l^−1^]. Individual changes in mitral TDI e’ wave in runners according to the results of NT-proBNP during the race [**(C)** NT-proBNP < 77 ng.l^−1^ versus **(D)** NT-proBNP > 77 ng.l^−1^].

**FIGURE 2:**
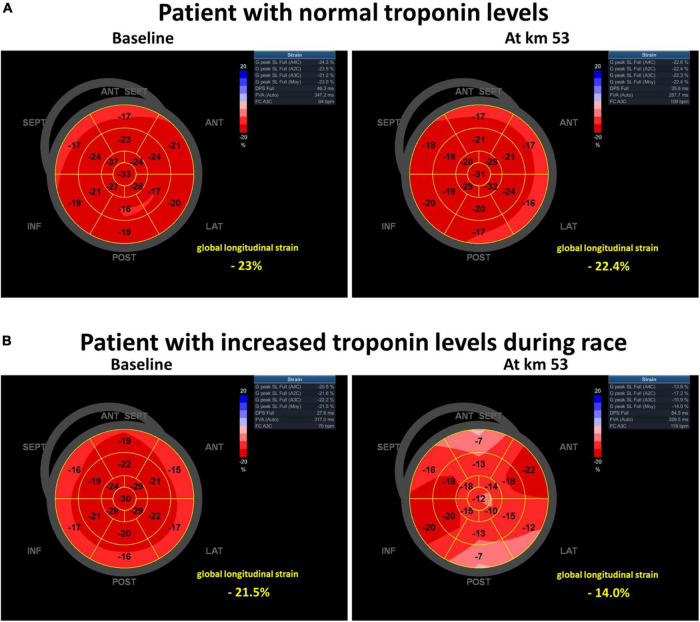
Pattern of global longitudinal strain (at baseline and at km 53), in a subject **(A)** with normal troponin levels and no left ventricular systolic dysfunction during the race and in a subject **(B)** presenting with increased troponin levels and left ventricular systolic dysfunction during race.

Diastolic echocardiographic abnormalities (E/A and mitral TDI e’ wave) were mainly observed among participants with NT-proBNP above the median value at the 21st km. As compared to baseline, mitral e’ wave was significantly decreased (–35%) in participants with NT-proBNP above the median value, whereas in participants with NT-proBNP values below the median value, no decrease in mitral e’ wave was observed (Table 4 and Figure 1). Similarly, GLS was also depressed among participants with T-troponin values above the median value, at the 53rd km (*p* = 0.01 at the 53rd km and *p* = 0.04 at arrival) whereas in participants with T-troponin values below the median value, no decrease in GLS was observed (Table 4 and Figures 1, 2). In addition, no significant association was observed between GLS, LVEF, or mitral S wave and NT-proBNP, as well as between mitral TDI e’ wave or mitral E/A and T-troponin.

**TABLE 4:** Relationship between echocardiographic parameters and cardiac biomarkers.

Variable	NT-proBNP < median value *N* = 10	NT-proBNP > median value *N* = 10	*P*
*Mitral E/A, m.s^–1^*			
Baseline	1.32 (1.23–1.56)	1.49 (1.32–1.63)	0.063
Km 21	0.99 (0.88–1.06)	1.01 (0.93–1.13)	0.006
Km 53	0.84 (0.78–0.90)	1.10 (0.94–1.12)	0.004
Km 80	0.84 (0.71–0.89)	1.03 (0.84–1.07)	0.006
*Mitral TDI e’ wave (m.s^–1^)*			
Baseline	0.16 (0.14–0.17)	0.17 (0.15–0.18)	0.062
Km 21	0.15 (0.12–0.18)	0.11 (0.10–0.12)	0.005
Km 53	0.15 (0.13–0.18)	0.11 (0.10–0.12)	0.004
Km 80	0.15 (0.14–0.18)	0.12 (0.11–0.13)	0.005
**Variable**	**T-troponin < median value *N* = 10**	**T-troponin > median value *N* = 10**	***P***
*Mitral S wave (cm.s^–1^)*			
Baseline	16 (15–18)	16 (14–17)	0.826
Km 21	17 (13–19)	17 (15–19)	0.658
Km 53	15 (14–18)	14 (13–15)	0.397
Km 80	14 (13–18)	14 (13–15)	0.165
*Global long. strain (%)*			
Baseline	–21.6 (–22.7 to –20.2)	–20.6 (–21.1 to –20.2)	0.464
Km 21	–22.0 (–22.9 to –21.6)	–23.0 (–23.8 to –20.6)	0.887
Km 53	–21.1 (–23.9 to –19.7)	–19.5 (–19.9 to –18.0)	0.012
Km 80	–20.1 (–21.0 to –19.9)	–18.3 (–19.2 to –17.9)	0.041
*LVEF (%)*			
Baseline	66 (61–69)	64 (62–68)	0.763
Km 21	65 (64–66)	66 (64–68)	0.057
Km 53	62 (61–65)	64 (60–65)	0.322
Km 80	62 (61–63)	57 (53–59)	0.001

Values are expressed as median value with interquartile range (1st quartile–3rd quartile).

Median value of NT-proBNP: 77 ng.l^–1^ and median value of T-troponin: 20 ng.l^–1^.

Global long. Strain, global longitudinal strain; and LVEF, left ventricular ejection fraction (%).

## Discussion

Our study is one of the first to investigate exhaustively echocardiographic and biomarker characteristics during long-distance trail running. The main results are: (1) significant differences in the systolic and diastolic functions were observed during the race, (2) mitral S wave and GLS were significantly decreased from the 53rd km to arrival, and all the systolic function parameters were significantly decreased at arrival, (3) mitral E/A velocity ratio and mitral TDI e’ wave were significantly decreased from the 21st to the 80th km, (4) a significant increase in BNP and troponin was observed during the race, and (5) a relationship was observed between BNP and echocardiographic diastolic parameters and between T-troponin and GLS during the race.

The benefits of a regular and moderate physical activity on health and life expectancy in both sexes and in the general population are recognized and are an integral part of recommendations to improve quality of life (31). A dose-response relationship between the amount of physical activity and associated reduction in all-cause mortality in the general population has been described (32). Thus, on-going physical activity may be a factor in reducing the risk of cardiovascular diseases (31). Over the last decades, long-distance running has been widely practiced by amateur runners, with an increase of approximately 5,200% in the number of ultra-endurance races between 1978 and 2008 run by amateur athletes (2). However, little is known about the immediate and long-term impact of prolonged exercise on the heart.

Left ventricular systolic function is preserved in participants presenting with athlete’s heart. However, despite normal GLS, reduced left ventricular ejection fraction may be observed at rest. Using exercise echocardiography, an improvement of left ventricular ejection fraction is observed, confirming an adaptive mechanism in line with Klenk et al. study results’ contrary to the abnormalities observed in an acute exercise among untrained participants (33). In a previous study (14), we observed the occurrence of LV systolic and diastolic dysfunctions during long-distance trail running. This process was acute and different from the well-known adaptive athlete’s heart. Interestingly, in this study, we performed serial echocardiographic examinations and biomarker analyses, and we observed an association between echocardiographic and biomarker parameters. In the participants, echocardiographic systolic LV dysfunction was associated with increase in troponin level, whereas echocardiographic diastolic LV dysfunction was associated with increase in NT-proBNP blood levels. However, all the participants did not develop systolic and diastolic dysfunctions: indeed, nearly half of them presented with a pattern of systolic and diastolic echocardiography and biological “burnout” (Table 4), suggesting that mismanagement of prolonged exercise could affect both diastolic and systolic functions by transient myocardial injury. During the serial analyses, we observed that biomarker modifications occur earlier during the race than echocardiographic parameters. Despite these acute abnormalities, all the participants performed annually an average of five long-distance running race each year, and no long-term impact was observed 10 years after the race, suggesting that the modifications are reversible (34). Moreover, the progressive increase in urea and creatinine at the 21st km suggests a potential acute kidney injury that occurred during the race even if we were not able to exclude a muscle contribution and/or hydration level influence.

The main limitation of the study is the small number of included participants. A single physician performed the echocardiographic examinations and biomarker analyses during the long-distance trail running with several 5-min “pit stops,” and it was technically impossible to add other participants in the study. It was also impossible to perform a multivariate analysis, because the small number of participants did not allow for meeting the validity conditions of a multivariate analysis and consequently, running speed, a possible confounding factor, could not be taken into account to assess the potential influence of exercise intensity on the observed cardiac abnormalities. Beyond this limitation, this study is the first to assess the echocardiographic and biological cardiac parameter kinetics during a long-distance running race with time-limited stops.

## Conclusion

In this study, we report an early decrease in diastolic echocardiographic parameters associated with increase in NT pro-BNP blood levels, and a decrease in systolic echocardiographic parameters later associated with increase in troponin blood levels. Blood parameter abnormalities occurred earlier than those of the echocardiographic parameters. Long-distance running race is associated with cardiovascular changes: a transient cardiac, systolic and diastolic, injury, but does not seem to be associated with a long-term health impact.

## Data availability statement

The raw data supporting the conclusions of this article will be made available by the authors, without undue reservation.

## Ethics statement

The studies involving human participants were reviewed and approved by the French Committee on public safety Paris Ile-de-France IV (Reference: 2014/07) and the National Heart Agency (Number EudraCT: 2014-A00205-42). The patients/participants provided their written informed consent to participate in this study. Written informed consent was obtained from the individual(s) for the publication of any potentially identifiable images or data included in this article.

## Author contributions

RJ and NM: conceptualization, data curation, writing – original draft preparation, supervision, and validation. RJ and HH: methodology. RJ: software. NM: visualization and investigation. RJ, HH, JA, AV, and NM: writing – review and editing. All authors contributed to the article and approved the submitted version.

## Footnotes

1
www.traildeparis.com

2
http://www.R-project.org
